# Prevalence and Associated Factors of Anal Incontinence at Six Weeks after Vaginal Delivery: A Cross-sectional Study at Three Teaching Hospitals in Addis Ababa, Ethiopia

**DOI:** 10.4314/ejhs.v34i3.6

**Published:** 2024-05

**Authors:** Kumasa Abdissa, Eyasu Mesfin, Kiflom Tesfaye

**Affiliations:** 1 Department of Obstetrics and Gynecology, School of Medicine, College of Health Sciences, Addis Ababa University; 2 Associate Professor of Obstetrics and Gynecology, REI, School of Medicine, College of Health Science, Addis Ababa University; 3 Assistant Professor of Obstetrics and Gynecology, MFM, School of Medicine, College of Health science, Addis Ababa University

**Keywords:** Incontinence, anal incontinence, perineal tear, vaginal delivery, Ethiopia

## Abstract

**Background:**

Anal incontinence is defined as the involuntary loss of fecal material or flatus. The reported prevalence at 6 weeks postpartum varies from 4% to 39%. It is associated with reduced quality of life, negative psychogenic effects and social stigma. This study was done to assess its prevalence at 6 weeks after vaginal delivery and identify the associated factors.

**Methods:**

This is a cross-sectional descriptive study. Data was collected using questionnaire adapted from International Consultation on Incontinence Questionnaire on Urinary Incontinence–Short Form. Data was analyzed using SPSS version 20.5.

**Result:**

The prevalence of anal incontinence at 6 weeks after vaginal delivery was 8.6%. The majority of the cases, 28 (84.8%), had only flatus incontinence. Participants of age group 20-35 years had significantly lower odds of having anal incontinence compared to those above age 35 (P < 0.05). The odds of having incontinence compared to spontaneous vaginal delivery was about 5 times higher for forceps (AOR= 4.93 (95%CI:1.48, 16.44)) and vacuum (AOR= 5.53 (95%CI:1.18, 25.96)) deliveries. Second stage of labor duration of >120 minutes had more than 4 times odds of developing incontinence compared to duration of <30 minutes (AOR= 4.79 (95%CI:1.01, 22.82)). Second degree perineal tear was the most significantly associated variable compared to those without tear (AOR= 12.31 (95%CI:3.89, 39.00)).

**Conclusion:**

The prevalence of anal incontinence at 6 weeks after vaginal delivery was 8.6%. Maternal age, mode of delivery, duration of second stage of labor and perineal tear were the significantly associated factors.

## Introduction

Anal incontinence (AI), defined as the involuntary loss of fecal material or flatus, ranges in severity from an occasional leakage of stool while passing gas to a complete loss of bowel control (1). The prevalence of AI varies depending on the definition used, the population studied and the study type. Because of embarrassment and the reluctance to seek medical help, the prevalence of AI is underestimated, and the real figures are unknown (2). The overall prevalence of AI in the population is reported to be approximately 8.3 % (2, 3). However, the reported prevalence of anal incontinence among women varies between studies from 2% to 25% (2-9). The reported prevalence of postpartum AI at 6 weeks postpartum varied from 4% in primiparous to 39% in multiparous (10). According to a study done in Nigeria in 2015, the cumulative prevalence rate was 13.5% for anal incontinence (9). Altered fecal continence has been reported in as many as 25% of primiparous women at 6 weeks postpartum (11).

AI is a major concern after delivery with Obstetric Anal Sphincter Injuries (OASIS) and occurs in approximately 50% of cases at long-term follow-up (7,9,12). It is associated with reduced quality of life, negative psychogenic effects and social stigma. Yet, many women do not report their symptoms or seek treatment (4,12). A study showed that women who reported anal incontinence at 4 months post-partum were more frequently depressed when evaluated subsequently at 12 months postpartum using Edinburgh Postpartum Depression Scale (EPDS) ≥ 10 or antidepressant use criteria. The reported depression rates were 36.0% and 23.3% in those with fecal incontinence and flatus-only incontinence groups, respectively, while it was only 14.8% in the continent women group (4).

The etiology of fecal incontinence is broad and typically divided into neurologic and non-neurologic causes. The most common causes among women are non-neurologic, particularly fecal incontinence after OASIS (12). Age, parity, prolonged second stage of labor (SSOL), episiotomy and instrumental vaginal delivery were significantly associated with increased risk of anal sphincter injury leading to postpartum AI (2,3). Forceps delivery was reported to increase the risk of fourth degree perineal tears compared with vacuum (13). AI risk is also increased with extended episiotomy, prolonged labor, macrosomia and dystocia. Additional factors include: Asian origin, short ano-vulvar distance, ligamentous hyper laxity and lack of expulsion control (14). Although cesarean delivery is said to be protective, recent studies have demonstrated that it is not totally so (15,16).

Only half of the cases of AI after childbirth can be attributed to OASIS. The other half may in part be caused by clinically unrecognized sphincter injury known as occult OASIS which could be visible on ultrasound. In observational studies, occult anal sphincter lacerations have been detected with endoanal ultrasound in 9 - 36%, 0 - 21% and 80 - 83% of women after spontaneous, vacuum and forceps deliveries respectively (5,6).

As the long-term success of primary sphincter repair in resolving anal incontinence is reported to be as low as 44%, it is important to reduce trauma to the perineum while attending deliveries (14). Primary prevention of OASIs includes appropriate maneuvers of SSOL, and if possible, avoidance of instrumental deliveries. Mediolateral episiotomy may also have a preventive role in high-risk OASIs deliveries (17). Secondary prevention is based on the training of skilled birth attendants in recognition and repair of OASIs (14).

Despite the anticipated magnitude of this public health problem, there is limited data on the prevalence of AI after childbirth in our country. Healthcare providers dealing with maternal care and delivery are in a unique position to identify women with AI because pregnancy, childbirth, OASIS, and pelvic floor dysfunction are important risk factors that contribute to fecal incontinence in women. Thus, the purpose of this study was to assess the prevalence of AI at 6 weeks after vaginal delivery and the associated factors in three selected teaching hospitals in Addis Ababa, Ethiopia.

## Methods

This is a cross-sectional descriptive study. It was done at Tikur Anbassa Specialized Hospital (TASH), Gandhi Memorial Hospital (GMH) and Zewditu Memorial Hospital (ZMH) in Addis Ababa, Ethiopia, from November 2020 to March 2021. Women who gave birth vaginally in the selected target hospitals at gestational age (GA) ≥28 wks or birth weight ≥1000 gm during the data collection period and consented to participate in the study were included. Women who were previously diagnosed with AI, had known underlying neurologic disorders, had diarrheal illness and Inflammatory Bowel Disease at the time of interview, and those admitted late in second stage with unknown duration of SSOL were excluded from the study.

Sample size was calculated using Cochran's formula. Considering the absence of previous data in Ethiopia, 50% proportion (p=.5 to achieve maximum variability) and the desired confidence level and precision of 95% and ±5%, respectively, were used to calculate the sample size. The resulting sample size considering 20% non-response rate was 460.

Total deliveries for two months prior to data collection in the study area were obtained from HMIS registration books for proportional allocation of study subjects among the three hospitals. Accordingly, 240, 122 and 98 subjects were allocated to GMH, ZMH and BLH, respectively. The study subjects then were selected using systematic sampling, including all odd numbers.

Data was collected using a structured questionnaire adapted from International Consultation on Incontinence Questionnaire on Urinary Incontinence-Short Form (ICIQ-B) and risk factors associated with anal incontinence mentioned in different literature. Data was collected by trained health providers and the principal investigator.

Data collection was done in two phases at two points i.e. before discharge and a follow-up interview through telephone call at 6 weeks postpartum. Consent was obtained from each participant at initial contact for both phases of data collection after providing them with adequate information on both phases of data collection. The information collected in the first phase included socio-demographic (age, marital status, religion, occupational status and level of education) and clinical (gravidity, parity, gestational age of the pregnancy, onset of labor, augmentation, presentation, duration of SSOL, episiotomy, spontaneous perineal tear and birth weight) characteristics. The second phase of data collection was done to assess for the presence and type of anal incontinence.

The collected data was coded, cleaned and analyzed using SPSS version 25 statistical software. A stepwise analysis was then conducted to explore for presence and strength of association between the independent variables and AI. Initially, descriptive and cross-tabulation with AI analysis of all independent variables was done for descriptive statistics and test of independence. Then, bivariate and multivariate regression analyses were implemented using AI as a dichotomous variable i.e. ‘yes’ and ‘no’.

Bivariate regression analysis was conducted for each independent variable with chi-square value of <0.2 on cross-tabulation for test of independence. Multiple regression analysis was then employed for those with P value of <0.25 on bivariate analysis to control the confounding effect amongst the variables. Odds ratio (OR) with their 95% confidence intervals were computed to identify the presence and strength of association, and statistical significance was declared at p < 0.05.

Ethical clearance was obtained from the Research and Publication Committee of the Department of Gynecology and Obstetrics, College of Health Sciences, Addis Ababa University. Permission was also obtained from the study facilities to collect data. Participation in the study was completely voluntary, and informed consent was acquired from every participant before participation. The phase two telephone interview was conducted at the participants' chosen time of the day to assure their privacy and confidentiality. Women with AI were counseled and linked to where urogynecology service was provided.

## Results

**Socio-demographic characteristics**: Out of the 460 participants included in the first phase, 384 mothers who responded to the second phase data collection were included in the analysis. This makes a response rate of 83.3%. The age of the study participants ranged from 18 to 41 with mean (SD) age of 26 (±4.5) years. The age groups 20-25 and 26-30 years accounted for 45.3% and 35.2% of the participants, respectively. More than 94% were married. More than half, 210 (54.7%), have completed primary education.

**Obstetric characteristics**: Half of the study participants, 50.5%, were primiparous. Para II was the second common parity and accounted for 27.6%. The gestational age of about two-third, 65.6%, of the participants was 37 – 41 weeks. The majority of the fetal presentations were vertex accounting for 96.1%. The duration of SSOL in 274(71.4%) was up to 60 minutes while in 24(6.3%), the duration was more than 120 minutes. The majority of the deliveries, 88.8%, were via spontaneous vaginal delivery (SVD). Instrumental deliveries accounted for 9.1% of deliveries. The birth weight in 353(92%) of cases was 2500-4000 grams. The stillbirth rate was 2.6%. Nearly half of the mothers, 181(47.1%), had episiotomy. A total of 83(21.6%) mothers had perineal tear while 10 of these mothers had episiotomy but developed second degree additional perineal tear separate from the episiotomy site (Table 1).

**Table 1:** Obstetric characteristics of study participants who delivered vaginally at the target facilities, Addis Ababa, Ethiopia, November 2020 – March 2021GC. (n=384)

Variables	Frequency (%)
Parity	
I	194 (50.5)
II	106 (27.6)
III	48 (12.5)
IV and above	36 (9.4)
Gestational age	
< 37 wks.	31 (8.1)
37 – 41 wks.	252 (65.6)
>41 wks.	75 (19.5)
Unknown	26 (6.8)
Presentation of fetus	
Vertex	369 (96.1)
Breech	8 (2.1)
Face	7 (1.8)
Onset of labor	
Spontaneous	341(88.8)
Induced	43(11.2)
Was labor augmented	
Yes	55 (14.3)
No	329 (85.7)
Duration of SSOL	
<30 min	120 (31.3)
31-60 min	154 (40.1)
61-90 min	56 (14.6)
91-120 min	30 (7.8)
>120 min	24 (6.3)
Mode of delivery	
SVD	341(88.8)
Forceps	22 (5.7)
Vacuum	13 (3.4)
ABD	8 (2.1)
Outcome of delivery	
Alive	374 (97.4)
Stillbirth	10 (2.6)
Birth weight	
<2500 gm	24 (6.3)
2500-4000 gm	353 (92)
>4000 gm	7 (1.8)
Perineal tear	
Yes	83 (21.6)
No tear	301(78.4)
Degree of perinea tear	
1^st^ degree	59 (71.1)
2^nd^ degree	24 (28.9)
Episiotomy	
Yes	181 (47.1)
No	203 (52.9)

*SSOL: second stage of labor, SVD: spontaneous vaginal delivery, ABD: assisted breech delivery*

**Prevalence of anal incontinence at 6 weeks after vaginal delivery**: In the present study, 33 mothers reported having anal incontinence. Hence, the prevalence of AI at 6 weeks after vaginal delivery was 8.6%. Majority of the mothers with AI, 28(84.8%), had only flatus incontinence whereas only 2 had fecal-only incontinence. Both flatus and fecal incontinence were reported in 3 of the participants.

**Psycho-social effects of AI**: Among the mothers who had AI, 5(15.5%) isolated themselves from social activities while 7(21.2%) felt sad due to their condition. Only two sought medical advice. Three of them (8.8%) also had additional involuntary leakage of urine.

**Factors associated with AI**: A stepwise analysis was conducted to explore for presence and strength of association between the selected independent variables and AI. Variables with P values of <0.25 in the bivariate logistic regression analysis were age, educational status, parity, mode of delivery, duration of SSOL and perineal tear. After adjusting for potential confounders in multivariate logistic regression analysis, four variables (age, mode of delivery, duration of SSOL and perineal tear) remained to be significantly associated with AI (P < 0.05) (Table 2).

**Table 2:** Psycho-social effects of anal incontinence among study participant mothers with the condition at the target facilities, Addis Ababa, Ethiopia, Nov. 2020 – March 2021GC. (n=33)

Variables	Frequency (%)
Pain during/after defecation	
Yes	2 (6.6)
No	31(93.4)
Leakage hamper relationship with husband	
Yes	0 (0)
No	33 (100)
Self-isolation from social activities	
Yes	5 (15.5)
No	28(84.5)
Feel sad due to condition	
Yes	7 (21)
No	26(79)
Seeking medical attention	
Yes	2 (6.6)
No	31(93.4)
Involuntary leakage of urine	
Yes	3 (9)
No	30(91)
Difficult to differentiation feces and gas in rectum	
Yes	1 (3)
No	32(97)
Postpone defecation for 15 min.	
Yes	23(69.7)
No	10(30.3)
Wear dippers	
Yes	3 (9)
No	30(91)
Did soil pants	
Yes	3 (9)
No	30(91)
Did soil the bed	
Yes	2 (6.6)
No	31(93.4)

Participants of age groups 20-25, 26-30 and 31-35 years had significantly lower odds of having AI compared to those above age 35 (AOR = 0.11 (95%CI:0.02, 0.51), 0.12 (95%CI:0.03, 0.55) and 0.09 (95%CI:0.02, 0.58), respectively. Forceps and vacuum deliveries carry about 5 times higher odds of having AI compared to SVD with AORs of 4.93 (95%CI:1.48, 16.44) and 5.53 (95%CI:1.18, 25.96), respectively. Duration of SSOL of >120 minutes had more than 4 times odds of developing AI compared to those with duration of <30 minutes (AOR= 4.79 (95%CI:1.01, 22.82)). Presence of 2nd degree perineal tear was the most significantly associated variable, with more than 12 times higher odds of developing AI compared to those without tear (AOR= 12.31 (95%CI:3.89, 39.00)). Mothers with 2nd degree tear included 10 mothers who had episiotomy but developed additional 2nd degree tear. Presence of first degree perineal tear, however, was not associated with AI (AOR= 1.74 (95%CI:0.52, 5.85)) (Table 3).

**Table 3:** Factors associated with anal incontinence after vaginal delivery at the target facilities, Addis Ababa, Ethiopia, November 2020 – March 2021 GC. (n=384)

Variables	Category	AI		COR (95% CI)	P Value	AOR (95% CI)	P Value

		Yes (%)	No (%)				
Age							
	< 20	3 (20)	12 (80)	0.55 (0.11, 2.86)	0.477	0.18 (0.02, 1.48)	0.110
	20 – 25	14 (8)	160 (92)	0.19 (0.06, 0.63)	0.007*	0.11 (0.02, 0.51)	0.005*
	26 – 30	8 (6)	125 (94)	0.14 (0.04, 0.50)	0.003*	0.12 (0.03, 0.55)	0.007*
	31 – 35	3 (6.5)	43 (93.5)	0.15 (0.03, 0.74)	0.02*	0.09 (0.02, 0.58)	0.011
	> 35	5 (31.2)	11 (68.8)	1		1	
Educational status							
	Uneducated	5 (16.7)	25 (83.3)	1		1	
	primary school	12 (5.7)	198 (94.3)	0.30 (0.09, 0.93)	0.037*	0.39 (0.09, 1.52)	0.173
	Secondary school	13 (14.1)	79 (85.9)	0.82 (0.27, 2.54)	0.734	0.72 (0.17, 3.01)	0.652
	College & above	3 (5.8)	49 (94.2)	0.31 (0.07, 1.39)	0.124	0.12 (0.02, 0.98)	0.047
Parity							
	I	17 (8.8)	177 (91.2)	0.48 (0.18, 1.32)	0.154	0.62 (0.16, 2.38)	0.485
	II	6 (57)	100 (94.3)	0.30 (0.09, 0.99)	0.050*	0.43 (0.10, 1.85)	0.258
	III	4 (8.3)	44 (91.7)	0.46 (0.12, 1.75)	0.251	0.51 (0.09, 2.69)	0.428
	IV and above	6 (16.7)	3 0(83.3)	1		1	
Mode of delivery							
	SVD	20 (5.9)	321 (94.1)	1		1	
	Forceps	7 (31.8)	15 (68.2)	7.49 (2.74, 20.45)	0.000*	4.93 (1.48, 16.44)	0.009*
	Vacuum	4 (30.8)	9 (69.2)	7.13 (2.02, 25.18)	0.002*	5.53 (1.18, 25.96)	0.030*
	ABD	2 (25)	6 (75)	5.35 (1.01, 28.22)	0.048*	1.92 (0.21, 17.46)	0.561
Duration of SSOL							
	< 30 min.	7 (5.8)	113 (94.2)	1		1	
	31 – 60 min.	13 (8.4)	141 (91.6)	1.49 (0.58, 3.86)	0.413	1.56 (0.50, 4.88)	0.442
	61 – 90 min.	5 (8.9)	51 (91.1)	1.58 (0.48, 5.23)	0.451	1.92 (0.48, 7.77)	0.359
	91 – 120 min.	3 (10)	27(90)	1.79 (0.44, 7.39)	0.419	1.18 (0.18, 7.59)	0.859
	≥ 120 min.	5 (20.8)	19 (79.2)	4.25 (1.22, 14.77)	0.023*	4.79 (1.01, 22.82)	0.049*
Perineal tear							
	1^st^ degree	5 (8.5)	54 (91.5)	1.46 (0.52, 4.09)	0.476	1.74 (0.52, 5.85)	0.369
	2^nd^ degree	10 (41.7)	14 (58.3)	11.23 (4.38, 28.78)	0.000*	12.31 (3.89, 39.00)	0.000*
	No tear	18 (6)	283(94)	1		1	

*AI: anal incontinence, SSOL: second stage of labor, SVD: spontaneous vaginal delivery, ABD: assisted breech delivery*.

* *P<0.05*

## Discussion

In the current study, the prevalence of AI at 6 weeks postpartum was 8.6%. This prevalence is lower than many prior reports from resource limited and developed countries. Studies done in Nigeria and South Africa reported significantly higher prevalence of AI at 6 weeks postpartum of 13.5% and 61.1%, respectively (9,18). Studies from developed countries, Canada and France, also reported higher prevalence of 25% and 14.4%, respectively (4, 19). However, it showed a higher prevalence than a Chinese study which reported 4% prevalence (20).

One possible reason for the low prevalence in the current study is under-reporting. The prevalence of AI is often under estimated as the majority of those having AI symptoms do not seek medical advice or intervention (1). In addition, some previous studies have identified increasing parity as a predisposing factor for AI (10, 21). In our study, more than half of the study participants gave birth for first time which possibly contributed to the lower prevalence of AI.

Anal incontinence is associated with reduced quality of life, and has physical and psycho-social effects (4,12). In the current study, 15.5% of the mothers who had AI reported to have isolated themselves from social activities because of their condition. Seven out of 33(21.2%) also reported that they felt sad. Only two of them visited health facilities to seek treatment. Prior studies have also reported similar findings. In the previously mentioned study in France, the percentage of depressed women was especially high among women with fecal incontinence (50.0%) but also higher in women with flatus incontinence (26.6%o) than in continent women (17.2%) (4). A study done in the US also showed significant psychological distress to be present in women with incontinence (22).

In the current study, four variables: age, mode of delivery, duration of second stage of labor and perineal tear were significantly associated with AI (P < 0.05). Maternal age of <35 carried a lower odds of developing AI. This result is similar to those reported by some prior studies suggesting that intrinsic factors may favor the occurrence of incontinences throughout gestation and increase the risk after delivery (9,10,23-25). According to a study from France, age over 35 years (OR: 6, 95% CI: 1.85, 19.45) was associated with an increased risk of AI regardless of the type of delivery compared to a group of women without AI (10). A study done by a pelvic floor research group in Spain reported age older than 35 years to be associated with higher risk of AI (Adjusted HR 1.7, 95% CI:1.0, 2.8) (25). Another prospective study on 525 women also reported age >30 years (AOR 4.60, 95% CI:1.11, 19.1) to be an independent risk factor for fecal incontinence at six weeks postpartum (24). The exact reason for increasing risk of AI with increasing age is not clear. Decreasing tissue elasticity with aging is considered to predispose to increased risk of obstetrical sphincter lacerations which subsequently increases the risk of AI (26).

Forceps and vacuum deliveries carried about 5 times higher odds of having AI compared to SVD with AORs of 4.93 (95%CI:1.48, 16.44) and 5.53 (95%CI:1.18, 25.96), respectively. This finding is in agreement with many prior studies (3, 9, 10, 19, 23, 27-29). Vacuum delivery may not be an independent risk factor as it is usually conducted for prolonged SSOL which by itself increases the risk (26, 30). However, with forceps delivery; the forceps adds to the presenting diameter of the fetus increasing the risk of lacerations to perineal muscles, pelvic floor, anal sphincter, and the surrounding fascia. Therefore, decision on using forceps for delivery needs caution considering the benefit and the risk. Besides if forceps is used to assist delivery, close monitoring for AI symptoms and timely pelvic floor rehabilitation training to prevent AI should be considered (30).

Duration of SSOL of >120 minutes had more than 4 times odds of developing AI compared to those with duration of <30 minutes (AOR= 4.79 (95%CI:1.01, 22.82)). This finding is comparable to the finding of a Nigerian study (9). The length of the first and second stages of labor was also found to be associated (P < 0.05) with the development of AI postpartum in an Israeli study (29). In another study done in Ireland, duration of SSOL beyond 60 minutes led to a 1.6-fold (95% CI:1.03, 2.6, P = .01) increased risk of symptoms (28).

Second degree perineal tear had twelve times more risk compared to those who do not have recognized perineal tear (AOR= 12.31 (95%CI:3.89, 39.00)). However, first degree tear was not associated with increased risk of AI while third and fourth degree perineal tears were not reported in this particular study. There is lack of studies on the association between first or second degree perineal tears and anal incontinence to compare with this study. Even though there was no recognized 3rd or 4th degree perineal tear in this particular study, there is a risk of under-diagnosis of OASIs in the labor ward, leading to under reporting of especially 3rd degree tears (14). AI in women without clinically diagnosed sphincter tear may also be due to occult sphincter injuries which need further ancillary tests such as endoanal ultrasound (5,31). A strong association between occult injuries and anal incontinence has been reported in prior studies (31,32).

Although episiotomy and second degree tears are similar in depth of involvement of perineal structures, second degree tears mostly occur along the median in the direction of anal sphincter. Hence, there is a high possibility of extension to involve the anal sphincter which could be missed during repair. Prior studies have reported increased risk of OASIS in median episiotomy which is similar to median second degree perineal tear (19).

In this study, episiotomy was not associated with AI. This is in agreement with many prior studies (27). In a systemic review of 12 studies that examined episiotomy as a risk factor for anal incontinence, only 2 reported significant associations between episiotomy and AI (33).

In conclusion, the prevalence of anal incontinence at 6 weeks after vaginal delivery was 8.6%. Maternal age, mode of delivery, duration of SSOL and perineal tear were the significantly associated factors. Thus, it is important to prevent perineal tear, instrumental delivery associated trauma and prolongation of SSOL at the time of delivery. The finding of association between second degree perineal tear and AI raises the possibility of misdiagnosing third degree tears and occult anal sphincter tears. Healthcare providers need to strictly assess for presence of anal sphincter involvement in presumed second degree tears. In addition, nationwide multicenter studies are recommended to determine a more representative prevalence and associated factors, and design effective preventive strategy.
